# Patients’ expectations of primary health care from both patients’ and physicians’ perspectives: a questionnaire study with a qualitative approach

**DOI:** 10.1186/s12875-024-02389-2

**Published:** 2024-04-24

**Authors:** Andreas Oster, Eivor Wiking, Gunnar H Nilsson, Christina B Olsson

**Affiliations:** 1 Barkarby Primary Health Care Center, Region Stockholm, Stockholm, Sweden; 2 Academic Primary Health Care Center, Region Stockholm, Stockholm, Sweden; 3 Department of Neurobiology, Care Sciences, and Society, Karolinska Institutet, Stockholm, Sweden; 4 Department of Neurobiology, Care Sciences, and Society, Division of Physiotherapy, Karolinska Institutet, Alfred Nobels Alle 23, Stockholm, 141 83 Sweden

**Keywords:** Patient expectations, Patient-centered care, Physician-patient relations, Primary health care

## Abstract

**Background:**

Patients’ ideas, concerns, and expectations are three important concepts in consultation techniques. Limited studies on these concepts include responses from both health care providers and care recipients of the same consultation. Highlighting both perspectives provides an increased understanding of the consultation. This study aims to explore the perspectives of patients and health care professionals about patients’ expectations of primary health care during consultations with primary care physicians and compare the two sets of perspectives.

**Methods:**

A cross-sectional study. Patients (*n* = 113) and physicians (*n* = 67) from five primary health care centers completed a questionnaire after planned consultations. Their responses to open-ended questions about patients’ expectations, from patients’ and physicians’ perspectives were analyzed with qualitative content analyses.

**Results:**

The patients expected a personal journey, through the primary health care system where they were the subject of interest. A journey, with ready access to a health care provider followed by a consultation with the physician, medical measures administered, their outcomes discussed, and a plan developed for continued health care. The physicians observed patients’ expectations to concern the responsibilities placed on primary health care where patients were the object of interest. Patients’ short-term expectations were described in a similar way by both patients and physicians. Patients expressed their long-term expectations as more personal and interpersonal whereas physicians observed them from a more professional and organizational standpoint.

**Conclusions:**

Patients and physicians have different views of what patients expect of primary health care. While patients’ short-term expectations were perceived by physicians, their long-term expectations were not. Patients expected more of a personal journey through the primary health care system while physicians observed patients’ expectations to concern the responsibilities placed on primary health care. Identifying and meeting patients’ expectations is an important part of patient-centered care, and a better understanding of patients’ expectations is needed to improve health professionals’ consultation skills.

## Background

The consultation between the patient and the health care professional has changed in structure throughout medical history. Modern health care has increasingly included the patient in his or her own care. This has led to health care education now including consultation courses with a patient-centered focus.

Patients’ ideas, concerns, and expectations (ICEs) are three important concepts in consultation techniques taught to health care professionals [1, 2]. Giving the patient room to express these early in the consultation is an important part of patient-centered care [3, 4].

Health care in Sweden and internationally has, during the 2000s, invested in increased patient centering in health care [5, 6]. There are differences in how ‘patient-centered care’ is internationally, and no clear consensus exists. Common factors, however, are that the care is described as including the individual patient and his or her relatives in coordinated care. The treatment is characterized by empathy, respect, and commitment where, among other things, the patient is given space to communicate his or her ideas, concerns, and expectations. This may form the basis for joint decision making by patients and health care professionals [7].

Patient-centered care has been shown to lead to improved health and a reduction in symptoms for the patients. Fewer misunderstandings during the consultation lead to a reduced risk of incorrect diagnosis and increased adherence to prescribed treatment. This in turn results in more efficient care, with a smaller number of diagnostic tests, examinations, and referrals, reduced unnecessary prescription of medication, and lowered long-term health care costs-term [8–10].

There are limited studies on patients’ ICEs that include responses from both health care providers and care recipients of the same consultation. Studying the difference between the patient’s and the physician’s perspectives of what has been expressed during the physician’s appointment provides a deeper insight into the meeting and may highlight important similarities and differences between both parties in the consultation.

In a previous study about patients’ ICEs, we invited physicians and patients to fill in a questionnaire after a planned consultation. A higher proportion of patients (87.8%) reported that their expectations had been met by the visit compared with what the physicians had observed (76.9%). The physicians in turn reported to a greater extent (30.8%) that the patients expected something more than the stated reason for the visit, which was expressed by only 3.8% of the patients [11].

Highlighting both physicians’ and patients’ perspectives thus provides an increased understanding of the consultation, which may contribute to increased knowledge and possible improvements in primary health care (PHC).

The main aim of this study is to explore patients’ expectations of PHC from the perspectives of both patients and physicians, respectively and comparatively. The specific aim was to find out what expectations patients express, what expectations physicians observe, and how the two sets of perspectives compare.

## Methods

This study is a part of a cross-sectional questionnaire study of patients’ ICEs in PHC. Details of the setting, sampling, and ethics can be reviewed in the previous paper [11]. The focus of this paper is the responses to open-ended question 15 of a questionnaire administered after a planned patient-physician consultation.

Two questionnaires were used: one for patients, which asked about their experiences, and one for physicians, which asked about their observations of the patients’ experiences. The questions were based on items in questionnaires used in earlier studies of patient-centeredness [12–17] and were revised, translated, and adapted to PHC in Sweden.

Patients were asked to describe their expectations of health care while physicians were asked a two-fold question. The first part addressed whether the physician had observed what the patients expected, response alternatives being ‘yes,’ ‘partly,’ ‘no,’ and ‘I don’t know.’ The second part consisted of an open-ended question asking for a more detailed description of the physician’s observations of patients’ expectations (Table 1).

**Table 1:** Open-ended questions to patients and physicians

Patients	Physicians
What are your expectations of continued care and treatment?	Has it emerged what the patient’s expectations are of the continued care and treatment? - yes - partly - no - I don’t know If so, regarding what?

The study included five PHC centers and two rehabilitation centers in northeast Stockholm. Data was collected from 1 February 2015 to 31 July 2015. In the current study, only data concerning the consultation with a physician is included. All physicians included were fully trained specialist physicians in family medicine. The population of the three municipalities represented in this study generally has a high socioeconomic status and educational level, and Swedish is the most commonly used language.

Included patients were Swedish-speaking adults booked for a consultation with a physician. The receptionists at the PHC centers gave oral and written information about the study prior to inclusion, highlighting the voluntary and anonymous nature of participation. Only patients attending planned consultations were included, not those attending acute care consultations. It was not possible to include acute care consultations because these are generally too short for both patients and professionals to have time to reflect on patients’ ICEs.

### Informed consent

was obtained from all subjects and/or their legal guardian(s) in case of minors (below 16 years of age). After obtaining informed consent, the receptionists provided two pseudonymous questionnaires with matching codes: one for the physician and one for the patient. The codes enabled the researchers to match the responses from the same consultation. After the consultation, patients and physicians were to separately complete their questionnaires and return them to the receptionist. To allow the participants time to reflect on their answers, the questionnaires could either be handed in or left in a sealed box. The completed questionnaires were kept in sealed boxes until they were collected by one of the researchers.

Included replies to question 15 came from both paired and unpaired questionnaires. In 40 consultations, questionnaires were filled out by both the patient and the physician. Additional responses from unpaired questionnaires came from 73 patients and 27 physicians. The total number of included participants was therefore 113 patients and 67 physicians (Table 2). The majority of respondents were women. Most patients who consulted a physician were ≥ 50 years old. The most common reasons for consultations with physicians were musculoskeletal, circulatory, and psychological problems. Questionnaires lacking responses to the actual questions e.g. describing experiences and satisfaction with previous health care or not expressing expectations on future health care, were excluded from this study.

**Table 2:** Gender and age distribution of respondents to question 15

Patients (*n* = 113)				Physicians (*n* = 67)		
**Age (yrs)**	Women (*n* = 76)	Men (*n* = 35)	Unknown (*n* = 2)	Women (*n* = 41)	Men (*n* = 26)	Unknown
20–49	21	7	–	10	11	–
50–70	35	15	–	31	15	–
> 70	17	12	1	–	–	–
Unknown	3	1	1	–	–	–

### Content analysis

The method chosen for analysis was qualitative content analysis. This is commonly used in education and nursing research. Qualitative content analysis assumes that reality is subjective and can be interpreted differently. This is important when discussing the trustworthiness of the results.

In content analysis, the analysis unit is the subject of the study which is divided into meaning units. The meaning units are condensed, interpreted, and aggregated. In this study, the aggregated levels were labeled ‘subthemes’ and the more abstracted levels ‘themes’ [18].

The analysis unit consisted of the detailed responses about the patients’ expectations of continued health care. The responses from patients and physicians were analyzed separately, establishing two different results. The results were then compared. Those subthemes that existed in both results were collected into a group of common subthemes. Those that were uniquely expressed by either participant group were separately grouped into patient responses, and physician responses. Finally, the three collections were each aggregated to find the common denominator in each group.

The results from the questionnaires were compiled, and preparatory content analysis was conducted separately by two members of the research group (AO and EW) as a basis for the subsequent group analysis. Here the separate analyses were compared and discussed together by all researchers until consensus was reached. The group analysis was repeatedly performed by the research group consisting of a physiotherapist with a PhD in physiotherapy and years of experience in primary health care, two specialist physicians (one of whom is a professor of general medicine and the other who has a PhD in general medicine), and one resident M.D.

The study was approved by the Regional Ethics Review Board in Stockholm, Sweden, Dnr 2014/ 1851-31. For more information about ethics see Freilich et al. [11].

## Results

Two distinct sets of themes emerged from the perspectives of the patients and physicians when describing the patients’ expectations of PHC. The patient themes had an inherent temporal order that followed the patients’ journey through the care process depending on which part of PHC was described. They concerned expectations of the health care providers, of the consultation, of the actions taken and their outcome, and of the plan for continued health care. The physicians expressed their perception of the patients’ expectations, and these themes had an abstract organizational order based on the meaning unit’s classification in subthemes that described the responsibilities of the health care professionals towards the patients. They also concerned the patients’ expectations of the structure of health care, the services it provides, and the endpoints of health care.

### Patients’ expectations of primary health care – a journey through the health care system

Five themes and 18 subthemes were identified from the patient data (Table 3). In the text below, the patient themes are ordered from one to five in order of their appearance in the patient’s journey through the health care system. The patients’ expectations begin with the contact with a health care provider, followed by the consultation with a physician. This leads to actions taken, followed by their outcome. Finally, the expectations concern the plan for continued health care. Up to three patient quotations are provided to illustrate each of the themes and subthemes expressed. Patients are identified by the patient number that is included with the quotes.

**Table 3:** Patients’ expectations of primary health care (PHC) – patient themes and subthemes

Themes	Health care provider	Consultation	Action	Outcome	Plan
**Subthemes**	AvailabilityProficiency	Approachability Communication Patient–physician relationship	Assessment Care planning Good care process Investigation Medical treatment Referral	Best possible results of care Explanations given Information Symptom relief	Continuity Follow-up Regular long-term follow-up

#### Expectations of the health care provider

##### Availability

Patients expect to get help and medical care when needed. This should be easily accessible, with short to reasonable waiting times; however, longer waiting times may also be experienced. Access to care through different channels is appreciated; however, telephone accessibility is in need of improvement.

Patient 4 (P4). ‘The waiting time is so long […].’;P15. ‘[…] easier telephone contact with a physician.’; P48. ‘[…] know that you can come urgently. […] good that you can book via the Internet […].’; P51. ‘[…] help when I seek care.’; P78. ‘[…] get an appointment quickly when necessary.’

##### Proficiency

The health care providers are expected to be professional and knowledgeable.

P53. ‘Professional and up-to-date …’.

#### Expectations of the consultation

##### Approachability

Patients expect to be treated well by a committed and responsive health care professional who takes their needs seriously and has a respectful approach.

P15. ‘More committed nurses. […]’; P55. ‘To be met respectfully.’; P82. ‘To be taken seriously and listened to …’; P92. ‘A positive, warm welcome […]’.

##### Communication

Good communication during the consultation is expected; good communication is also seen as an integral part of the follow-up process.

P68. ‘Continued good communication and monitoring of the problem.’

##### Patient–physician relationship

Patients expect to be able to trust their physician and have a good relationship with him or her. Some also expressed the expectation that an already established, functioning patient–physician relationship may be difficult to retain.

P17. ‘To have good contact with my physician […]’; P34. ‘If all physicians were as responsive and competent as mine, health care would be perfect. However, I have previously had many bad experiences with misunderstanding, stressed, unresponsive, and even downright rude physicians. […]’.

#### Expectations of actions

##### Assessment

Patients expect to get a physician’s assessment of any health issue(s) or problem(s).

P93. ‘To get a survey of the problem.’

##### Care planning

The physician and patient are expected to plan the patient’s health care together, using the physician as a professional guide on how best to proceed.

P90. ‘I hope CBT [cognitive behavioral therapy] will help me. A common question you get is: “How do you want to proceed?” And that’s exactly what you need help with.’; P108. ‘To get suggestions/ideas on how to alleviate or cure my problems …’.

##### Good care process

A high standard of health care, being satisfied, and getting help with current health issues while also having them documented is expected by the patients. A continued high level of service, and a continuation of service, is also expected with intact health care delivery.

P26. ‘… that they note my condition today and move on to the next step at the next visit, if the condition is the same.’; P43. ‘To continue as now, with no downsizing to try to save money and staff …’; P61. ‘… that it is documented …’.

##### Investigation

Patients expect the health care professional to investigate new and lapsing symptoms.

P11. ‘To investigate what causes my issues and [to take] measures …’; P45. ‘That you discover any faults and causes.’; P52. ‘[…] getting [an] investigation of the heart.’

##### Medical treatment

The health care provider is expected to initiate treatments, change medications or their dosage, and decide whether or not to terminate or continue a treatment.

P8. ‘Continued prednisolone treatment […]’; P54. ‘[…] relevant treatment.’; P63. ‘Change the medicine or stop the treatment.’; P82. ‘[…] receive immediate treatment.’

##### Referral

Patients expect to have consultations with specialists or experts, and to get an appointment with them within reasonable time, as well as having continued contact with secondary health care.

P2. ‘… awaiting an examination and information from the eye specialist …’; P10. ‘… that the waiting time for referral is not too long.’; P58. ‘Not to be dropped [dismissed] by those I am referred to.’

#### Expectations of outcome

##### Best possible results of care

These expectations range from being completely cured to getting the maximum improvement of function possible.

P87. ‘… to be completely restored.’; P101. ‘[… to] be as good as possible, so I can continue walking, which is a part of my life.’; PÖ31. ‘… that I should feel better.’

##### Explanations given

The cause of patient symptoms is expected to be found and explained.

P25. ‘… to get further answers on the cause of my dizziness.’; P65. ‘… that I will find out what causes my problems.’

##### Information

Being informed of results from investigations and the diagnosis.

P18. ‘Response to further investigation.’; P99. ‘… that it is clarified what the pain/’gravel’ in my feet [is].’

##### Symptom relief

Patients expect to have their symptoms reduced or removed if possible.

P23. ‘To get pain relief and help with breathing.’; P62. ‘I want to get rid of the pain!!! But I suspect I may continue to live with it.’

#### Expectations of the care plan

##### Continuity

Being able to see the same physician throughout the care process is one of the patients’ expectations.

P9. ‘[I] think it is important to be able to see the same physician.’; P60 ‘… that I get an appointment with the same physician in the event of a revisit.’

##### Follow-up

Patients expect that they get a new appointment concerning their condition where they will get results from current investigations as well as an assessment of their response to treatment.

P83. ‘… as good a follow-up of my problems as today. Get a new appointment, which we agreed on in autumn.’; P89. ‘… that the follow-up for the fatigue continues, with physician visits and X-rays for a long time to come.

##### Regular long-term follow-up

General checkups and follow-up for chronic conditions are expected at regular intervals.

P24. ‘Continuous (annual) follow-up of health.’; P88. ‘… that you are called to your family physician once a year for a general checkup.’

### Physicians’ observations of patients’ expectations – responsibilities of primary health care towards patients

Three themes and 15 subthemes were identified from the physician data (Table 4). The themes relate to health care – its structure, processes and services, and the endpoints of PHC. The subthemes describe the patients’ observed expectations of these. Physician quotations are provided to exemplify the themes and subthemes expressed below. The physicians are identified by the physician number that is included with the quotes.

**Table 4:** Physicians’ observations of patients’ expectations – physician themes and subthemes

Themes	Structure of health care	Services of health care	Endpoints of health care
**Subthemes**	Availability Approachability Good health care	Care planning Certificates Follow-up Guidance Information Investigation Medical treatment Referral Rehabilitation	Better health Good health Good health care results

#### Structure of health care

##### Availability

Health care providers are expected to be available when the patient has a medical need. There is also patient concern that these expectations will not be met.

Physician 6 (PH6). ‘… that she [the patient] can come when she has problems.’; PH18. ‘[The patient is] fearful of not getting help.’

##### Approachability

Patients expect to be given the opportunity to explain their situation and symptoms including sending information to the health care provider beforehand.

PH16. ‘The patient needs to explain and describe [his or] her situation and symptoms. [He or] she can also send letters before the visit with information.’

##### Good health care

Health care is expected to remain at a good standard.

PH21 and PH49. ‘Continued good care.’

#### Services of health care

##### Care planning

The patient expects to develop a plan together with the physician.

PH40. ‘… that we set up a plan.’

##### Certificates

Health care professionals are expected to write medical certificates as required by their patients.

PH34. ‘[…] certificates for travel this autumn.’

##### Follow-up

Health care providers are expected to schedule revisits for patients, give feedback, take new blood samples, provide different methods of follow-up, and arrange regular visits for chronic diseases.

PH2. ‘Planned follow-up.’; PH5. ‘Feedback.’; PH7. ‘… call if she doesn’t get well. Get test results. Try a treatment.’; PH20. ‘Continued checkups.’; PH27. ‘Being healthy and just having to come for annual checkups.’; PH29. ‘… that we call the patient for follow-up.’

##### Guidance

Health care providers are expected to guide patients through the care process.

PH1. ‘… thoughts on colonoscopy.’; PH36. ‘… discussed planned X-ray and colonoscopy.’

##### Information

Patients expect to be informed of test results, their method of delivery, to be given relevant information, and to be reassured when results are benign.

PH15. ‘[The patient] wants test results.’; PH42. ‘… how the results of the examination will be delivered.’; PH55. ‘Correspondence.’; PH59. ‘Explanation that [the condition is] not dangerous.’

##### Investigation

Health care professionals are expected to investigate symptoms and perform specific investigations.

PH9. ‘MRI [magnetic resonance imaging].’; PH31. ‘Continued investigation.’; PH32. ‘Symptoms, cause, blood sampling.’; PH47. ‘[The patient] wondered if the wrist was fractured and, if it was, [would need to receive] treat[ment].’

##### Medical treatment

Patients expect to receive treatment – pharmaceutical, psychological, and surgical.

PH4. ‘[The patient] wants to get rid of the lesion.’; PH8. ‘Surgery of lump.’; PH13. ‘Internet treatment.’; PH24. ‘New medication.’; PH37. ‘How long to take medication.’; PH50. ‘Help reduce cortisone to eliminate side effects.’

##### Referral

Patients expect to be referred to other specialists in secondary health care when needed.

PH17. ‘Referral to an asthma physician.’; PH35. ‘Wants to meet a dermatologist, take [a] test for venereal disease […]’.

##### Rehabilitation

Health care providers are expected to support their patients to return to work.

PH19. ‘Support in returning to work.’; PH52. ‘Support for rehabilitation, returning to work.’

#### Endpoints of health care

##### Better health

Patients expect that their health will improve so they can return to normal life.

PH56. ‘The stomach will improve enough so the patient can get a life [again].’

##### Good health

Health care providers are expected to provide services for good health for their patients.

PH27. ‘Being healthy and just having to come for annual checkups.’

##### Good health care results

Patients expect to have good results from their contact with health care.

PH10. ‘Implicitly remove [the] pat[ient]’s pain …’.

### Patients’ and physicians’ perspectives of patients’ expectations – shared and unique subthemes

While the patient and physician themes differed, their subthemes both coincided and diverged (see Table 5). Eight subthemes concerning the patients’ expectations were expressed by both patients and physicians. They included the approachability as well as availability of health care. According to both parties the patients expected care planning and follow-up of care. The patients expected to be kept informed during the care process, along with taking part in investigations and medical treatments, as well as receiving referrals to secondary health care.

The commonly expressed patients’ expectations fell within a shorter time frame centered around the consultation between the patient and the physician, and the activities that followed.

### Unique subthemes

Ten subthemes were uniquely expressed by patients. These concerned expectations regarding the health care professional’s proficiency and the patient–physician relationship, which was expected to have a good care process, communication, continuity, with regular follow-ups. Also, the patients expected the assessment by the physician to include an explanation of symptoms while also expecting the best possible care results or, at least, symptom relief.

Six subthemes were uniquely expressed by the physicians. These subthemes were related to expectations of the health care system as a whole, including good health care and good, or improved, health for the patient. The unique physician subthemes also included rehabilitation, medical guidance, and the issuance of certificates.

**Table 5:** Patients’ expectations of primary health care (PHC) – comparison of patient and physician subthemes

Patient subthemes	Shared subthemes	Physician subthemes
Assessment Best possible care results Communication Continuity Explanation Good care process Patient–physician relationship Proficiency Regular follow-up Symptom relief	Approachability Availability Care planning Follow-up Information Investigation Medical treatment Referral	Better health Certificates Good health Good health care results Guidance Rehabilitation

## Discussion

### Summary of main findings

To our knowledge, this is the first study that explores patients’ expectations of PHC from the perspectives of both patients and physicians.

The patients expected a personal journey through the PHC system where they were the subject of interest. The physicians observed patients’ expectations to concern the responsibilities placed on PHC where patients were the object of interest.

Patients’ short-term expectations were described in a similar way by both patients and physicians. Patients expressed their long-term expectations as more personal and interpersonal whereas physicians observed them from a more professional and organizational standpoint.

### Patients’ expectations of primary health care

The open-ended prospective question on patients’ expectations provided a broader view of what patients expect throughout their contact with PHC. This facilitated the interpretation of the themes, which we summarized as the patients’ journey through the health care process.

Our results show that patients expect to be at the center of the care process both in the short term and in the long term. This is in line with our expected results and corroborates well with our knowledge of patient-centered care [7].

Unexpectedly, the long-term expectations of the patients differed from the physicians’ understanding of patients’ expectations. Patients solely expressed the expectation of a continued and regular, well-established relationship with their physician. They expected the physician to proficiently make an assessment of their health issue, to provide an explanation, and to achieve relief of symptoms and the best possible care results, also in the long term. These expectations on individual clinicians and health outcomes have previously been reported to be two important aspects of patients’ expectations [19–22]. Fulfilling these two expectations may increase the level of trust felt by patients, which may have positive effects on the health outcome [23, 24].

### Physicians’ observations of patients’ expectations

When asking the physicians about their observations of patients’ expectations, we gained valuable insights into their perspective, and into what they observed and how they observed it. This facilitated the interpretation of the themes, which portray an organizational and professional viewpoint, focusing on health care delivery and the responsibilities of PHC towards the patients.

The objective of PHC in Sweden is to achieve good health and health care on equal terms for the entire population, as defined by medical legislation [25]. Our results indicate that physicians in PHC in Sweden view some of their patients’ expectations from this professional perspective, where the patient and his or her health is the objective of health care.

Physicians did observe the short-term expectations of the patients on a group basis. However, they viewed the long-term expectations of the patients as relating to the responsibility of the health care system and its goals rather than the patients’ goals. In addition, physicians expressed expectations that concerned work-related tasks such as issuing certificates, providing medical guidance, and ensuring rehabilitation, which were not expressed by the patients.

### Comparing perspectives of patients’ short- and long-term expectations

Our results show that, today, during consultations with a physician in Sweden, patients’ short-term expectations are more successfully perceived by the physician. This may show the effects of the increased training of health care workers in patient-centeredness during the last decades [26].

The difference in perspective of patients’ long-term expectations between patients and physicians can be seen as a natural division between the different roles both sides have. While patients’ experience is affected by their medical condition from onset until the present, the physicians only get a glimpse of the patients’ experience during the brief consultations available in PHC while they also need to meet the expectations of the PHC organization they are working for.

In the present study, the patients expected more of a personal journey through the primary health care system while physicians observed patients’ expectations to concern the responsibilities placed on primary health care. In addition, the physicians viewed the patients’ long-term expectations as more related to the goals of the health care system than the patients’ goals. A review study [7] found that the goal of patient-centered care is a functional life while that of person-centered-care is a meaningful life. Adopting a more person-centered approach where the patient is seen as a person with needs and preferences beyond just the medical perspective [27, 28] might help to observe patients’ long-term expectations and goals. It has been suggested that implementing routines like eliciting the patient’s narrative, and documenting shared goals in partnership between the patient and the professional could be a way forward towards person-centered care [28, 29].

How, then, is the patient’s health and the health care affected when a physician has no observations or understanding of a patient’s long-term expectations? The personal expectations of a patient would go unseen and would be replaced by the general obligations of the health care system. The differing goals of two different parties would create barriers to patient-centered care, especially for more complex patients with multi-morbidity [30].

A question to be asked in this context is, what may have caused a lack of physicians’ observations of the patients’ long-term expectations in the past? An increased focus on economic management of the health care system may have gradually replaced the governance of the medical profession in defining the goals of health care, and got them to align their perspective with the overarching goals of health care management. Learning the patients’ long-term expectations may be an inherent challenge to the consultation method itself. During consultations, because of the brief time available to assess the patient’s current condition, there is the risk of focusing on the patient’s short-term expectations [31].

The answer would most likely involve both the management of the health care system, and the health care professions; any solution would need to consider both levels. Policy makers could offer grants and financial incentives to reward effective patient-centered communication in PHC [32] and improved education in patient-centered care could increase the skill and experience of health care professionals.

### Methodological considerations

The questionnaires used in this study asked the respondents about patients’ expectations of PHC. Since there were two types of respondents, patients and physicians, two different datasets were obtained, which needed to be analyzed separately. During content analysis, there is a risk that when analyzing one dataset first, this affects the analysis of the second dataset to be analyzed. To avoid this, we specifically aimed to perform the content analysis as two separate tasks. Any comparative effort was avoided until both analyses were completed.

There were more respondents in the patient group (*n* = 113) than in the physician group (*n* = 67). This may have caused the patient group to be perceived as expressing a higher number of unique themes that were not expressed by physicians.

The question asked concerned patients’ expectations of continued health care; however, because of the open-ended nature of the question, replies also included expectations in both present and past tense. The research group chose to include expectations in present tense as these were expected to continue into the future tense. Experiences with previous health care and whether or not the respondents had been satisfied in the past were excluded from the content analysis.

Some replies expressed no expectations on future health care. Since the intention of these replies is hard to discern, they were likewise excluded from the content analysis.

Short answers may provide little information and give rise to the risk of misinterpretation. While some interpretation is expected in content analysis, care is needed to remain close to the original data in the analysis. Working in a research group with different backgrounds as health care professionals and reaching consensus helped to minimize this risk.

Sometimes the sub-themes were difficult to distinguish and some of them overlap. We have tried to make the differences distinct, but the method involves a degree of interpretation that is unavoidable. However, different levels may be distinguished such as the individual level in “best possible care results”, the more diagnostic level in “symptom relief” and “good care results” and the general improvement as in “good health”. The role of primary care and family medicine is to see the whole but also the specific parts.

Since the questionnaires were filled out after a planned PHC consultation, the results should be interpreted accordingly and should not be generalized to other health care situations. Knowledge of a patient’s prior diagnosis would affect the expectations of both the patient and the physician.

The method of content analysis is not tailor-made for comparing two qualitative datasets. The comparative analysis is described in the Method section for increased scrutiny.

Most respondents were women aged ≥ 50 years with musculoskeletal, circulatory, and psychological problems and the study included both publicly and privately run primary care units. The described population is common in primary health care [33–37] which adds to the transferability of the results. However, the included care units are localized in an area with higher socioeconomic status compared to the country average, which may prevent transferability.

When taking multiple perspectives into account while examining a subject involving multiple parties you gain insights with greater trustworthiness. In addition, our research group consisted of different professions with different levels of clinical and academic experience, further improving the trustworthiness of the interpretation of results.

### Implications for health care and research

This study reveals a deeper understanding of the type and individual centeredness of the patients’ expectations and therefore has several implications for the delivery of patient-centered PHC. Applying our findings in clinical practice may improve adherence to treatment and help to avoid overdiagnosis and overtreatment as well as increase understanding of the patients’ long-term expectations.

It can be challenging to perceive a patient’s long-term expectations during a consultation. Our findings underline the importance of identifying the patients’ long-term expectations to support patient centeredness and to distinguish these expectations from management’s expectations put on the medical profession.

The positive effects of continuity in health care are well established [38]; however, the expectation for continuity and regularity of follow-ups may be difficult to meet. A personal care plan for the patient would be a tool with which to provide a form of continuity between different care providers.

While an assessment is an inherent part of a consultation, an adequate explanation for a patient’s symptoms is not always available. Extra care may be needed to communicate the assessment and, if possible, give a likely explanation for the symptoms to improve patient centeredness [39, 40].

We recommend further studies on the possible benefits of determining patients’ long-term expectations of their health care as well as optimal methods for achieving this task and studying the phenomenon of expectations in order to improve patient-centered care.

## Conclusion

Patients and physicians have different views of what patients expect of PHC. While patients’ short-term expectations were perceived by physicians, their long-term expectations were not. Physicians did not know what patients expected of PHC in the long term. Since finding out the patient’s expectations is an important part of patient-centered care, a better understanding of patients’ expectations is needed to improve consultation skills. Such an understanding can be both used in the professional education of physicians and implemented in clinical practice.

## Data Availability

The data (pseudonymized answers to the open-ended question no 15 in the questionnaire) analyzed during the current study are available from the corresponding author on reasonable request.

## Abbreviations

ICE: Ideas Concerns and Expectations
M.D.: Medical Doctor
P: Patient
PH: Physician
PHC: Primary Health Care
